# Chikungunya and Zika Virus Cases Detected against a Backdrop of Endemic Dengue Transmission in Vietnam

**DOI:** 10.4269/ajtmh.16-0979

**Published:** 2017-04-10

**Authors:** Nguyen Than Ha Quyen, Duong Thi Hue Kien, Maia Rabaa, Nguyen Minh Tuan, Tran Thuy Vi, Le Van Tan, Nguyen Thanh Hung, Ha Manh Tuan, Ta Van Tram, Nguyen Le Da Ha, Han Khoi Quang, Nguyen Quoc Doanh, Nguyen Van Vinh Chau, Bridget Wills, Cameron P. Simmons

**Affiliations:** 1 Oxford University Clinical Research Unit, Wellcome Trust Major Overseas Programme, Hospital for Tropical Diseases, Ho Chi Minh City, Vietnam; 2 Nuffield Department of Medicine, Centre for Tropical Medicine, University of Oxford, Oxford, United Kingdom; 3 Children’s Hospital No. 1, Ho Chi Minh, Vietnam; 4 Children’s Hospital No. 2, Ho Chi Minh, Vietnam; 5 Tien Giang Hospital, My Tho, Vietnam; 6 Dong Nai Children Hospital, Bien Hoa, Vietnam; 7 Binh Duong Hospital, Thu Dau Mot, Vietnam; 8 Long An Hospital, Tan An, Vietnam; 9 Hospital for Tropical Diseases, Ho Chi Minh, Vietnam; 10 Department of Microbiology and Immunology at the Doherty Institute, University of Melbourne, Parkville, Australia

## Abstract

Between 2010 and 2014, four chikungunya and two Zika virus infections were identified among 8,105 febrile children in southern Vietnam. Zika viruses were linked to French Polynesian strains, chikungunya to Cambodian strains. Against a backdrop of endemic dengue transmission, chikungunya and Zika present an additional arboviral disease burden in Vietnam.

## INTRODUCTION

The emergence of the Zika virus (ZIKV) in the Western Pacific and Latin America, followed by the World Health Organization (WHO) declaration of a global public health emergency, has brought renewed attention to this and other epidemic arboviral infections. ZIKV is a flavivirus, whereas chikungunya virus (CHIKV) is an alphavirus. Both viruses are transmitted to humans by *Aedes* spp. mosquitoes. Clinical signs and symptoms of CHIKV and ZIKV infections are similar to those of dengue, particularly in the first few days of illness when the symptoms are nonspecific, including but not limited to fever, arthralgia, maculopapular rash, muscle and joint pain, malaise, and headache.^1^

There are three genotypes of CHIKV: Asian, West African, and East/Central/South African (ECSA). The Asian genotype is thought to have circulated in southeast Asia for many decades without ever posing a perceivable public health problem. More recently, one lineage of the CHIKV ECSA genotype, the Indian Ocean Lineage (IOL), emerged from east Africa and seeded large epidemics in the Indian Ocean region, and later in Europe and Asia.^2^^–^^5^ Sporadic cases of illness caused by the CHIKV IOL were first detected in Cambodia in 2011.^5^ In neighboring Vietnam, no virologically confirmed cases of CHIKV infection have been described.

ZIKV has also evolved into three distinct genotypes of similar geographic distribution: West African (Nigerian cluster), East African (MR766 prototype cluster), and Asian.^6^ ZIKV was first detected in humans in 1954 but has recently emerged as a global threat to public health^7^^,^^8^ because it can be an infectious teratogen to the unborn fetus and an occasional cause of Guillain–Barré syndrome in adults.^9^^,^^10^ Since 2015, 67 countries have reported outbreaks (WHO situation report, October 27, 2016; http://www.who.int/emergencies/zika-virus/situation-report/27-october-2016/). Prior to 2016, only sporadic ZIKV infections have been reported in southeast Asia; sequence analysis shows that historical southeast Asian viruses cluster within the Asian ZIKV lineage.^7^^,^^11^ In Vietnam, the first two confirmed cases of locally acquired ZIKV infections were reported in southern Vietnam in April 2016 (http://www.who.int/csr/don/12-april-2016-zika-viet-nam/en/).

Against a backdrop of seasonally hyperendemic dengue transmission, this retrospective study sought to understand the burden and trajectory of CHIKV and ZIKV infections in Vietnam from 2010 to 2014.

## THE STUDY

This was a retrospective diagnostic study. The study population was children from 1 to 15 years of age with fever of less than 72 hours and clinical symptoms that suggested to the attending physician that dengue could be a possible diagnosis. Study participants were enrolled at the outpatient clinics of seven hospitals in southern Vietnam between October 2010 and December 2014. The detailed inclusion and exclusion criteria and study procedures are described elsewhere.^12^ Reverse transcription polymerase chain reaction (RT-PCR) tests for ZIKV^13^ and CHIKV^14^ were performed on stored frozen plasma samples from study participants that were tested negative in a dengue virus (DENV) RT-PCR assay that has been described extensively elsewhere.^15^ Virus surveillance was only carried out on stored specimens from patients who had consented to having their samples used for future research. The study protocol was reviewed and approved by the institutional review boards of the seven hospitals and the Oxford Tropical Research Ethical Committee (OxTREC 592-16).

## RESULTS

During the study period, 8,105 participants were enrolled and 2,203 dengue cases identified on the basis of direct virological confirmation (non-structural protein 1 or RT-PCR positive) or IgM seroconversion (Panbio IgM capture enzyme-linked immunosorbent assay) as described previously.^12^ Among all DENV PCR–negative samples (6,037), including those showing serological evidence of recent DENV infection, a total of 5,617 frozen acute plasma samples (collected within 72 hours of fever onset) were of adequate sample volume for CHIKV and ZIKV testing. Of the 5,617 samples tested, four CHIKV (0.07%) and two ZIKV (0.035%) cases were identified.

### Clinical manifestations.

**Figure 1.**
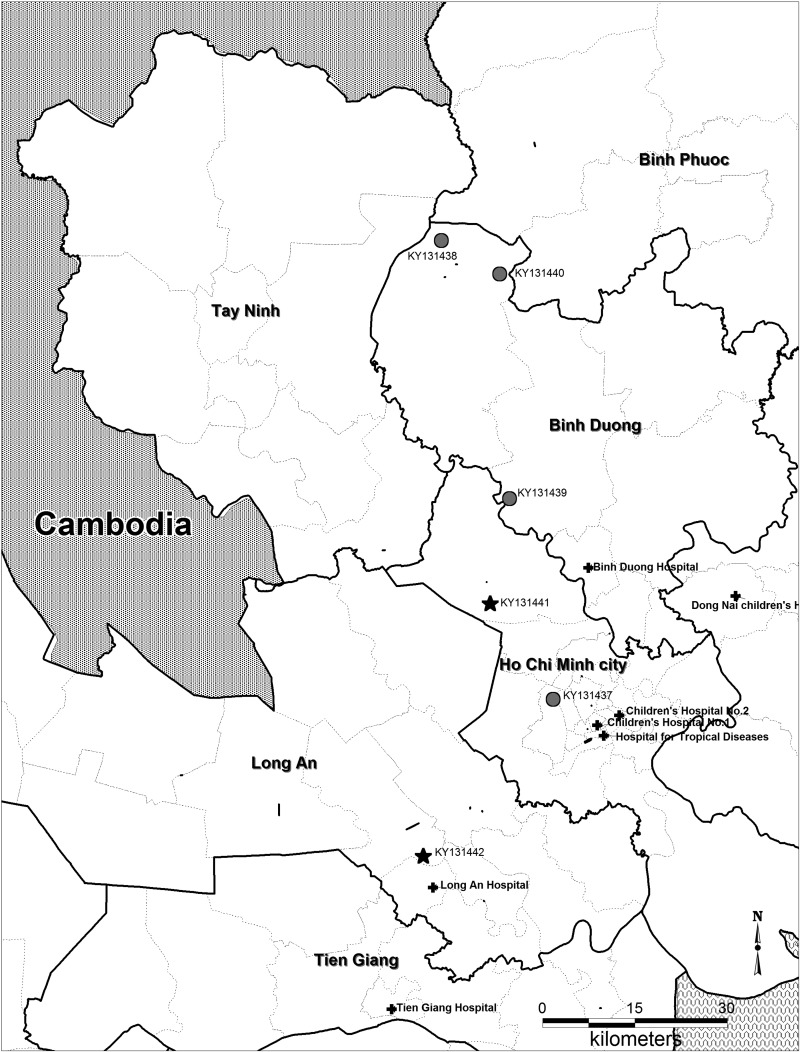
Residential location of the study patients and study hospitals. Locations of the chikungunya and Zika cases are shown with black circles and stars, respectively; hospitals are shown as black crosses. Two chikungunya cases were located approximately 20 km from the border with Cambodia.

All four CHIKV cases were detected between August and November 2012—one case lived in Ho Chi Minh City and three in Binh Duong Province (Figure 1

). Three of the infections were relatively mild, with fever, rash and mild to moderate systemic symptoms reported. One case, a 13-year-old boy, was briefly hospitalized. All patients made unremarkable recoveries.

The two ZIKV infections were identified in a 12-year-old from Long An Province, and a 4-year-old from Cu Chi District, Ho Chi Minh City, in January and June 2013, respectively. The 12-year-old was briefly hospitalized. Both children recovered fully.

### Viral genetics.

**Figure 2.**
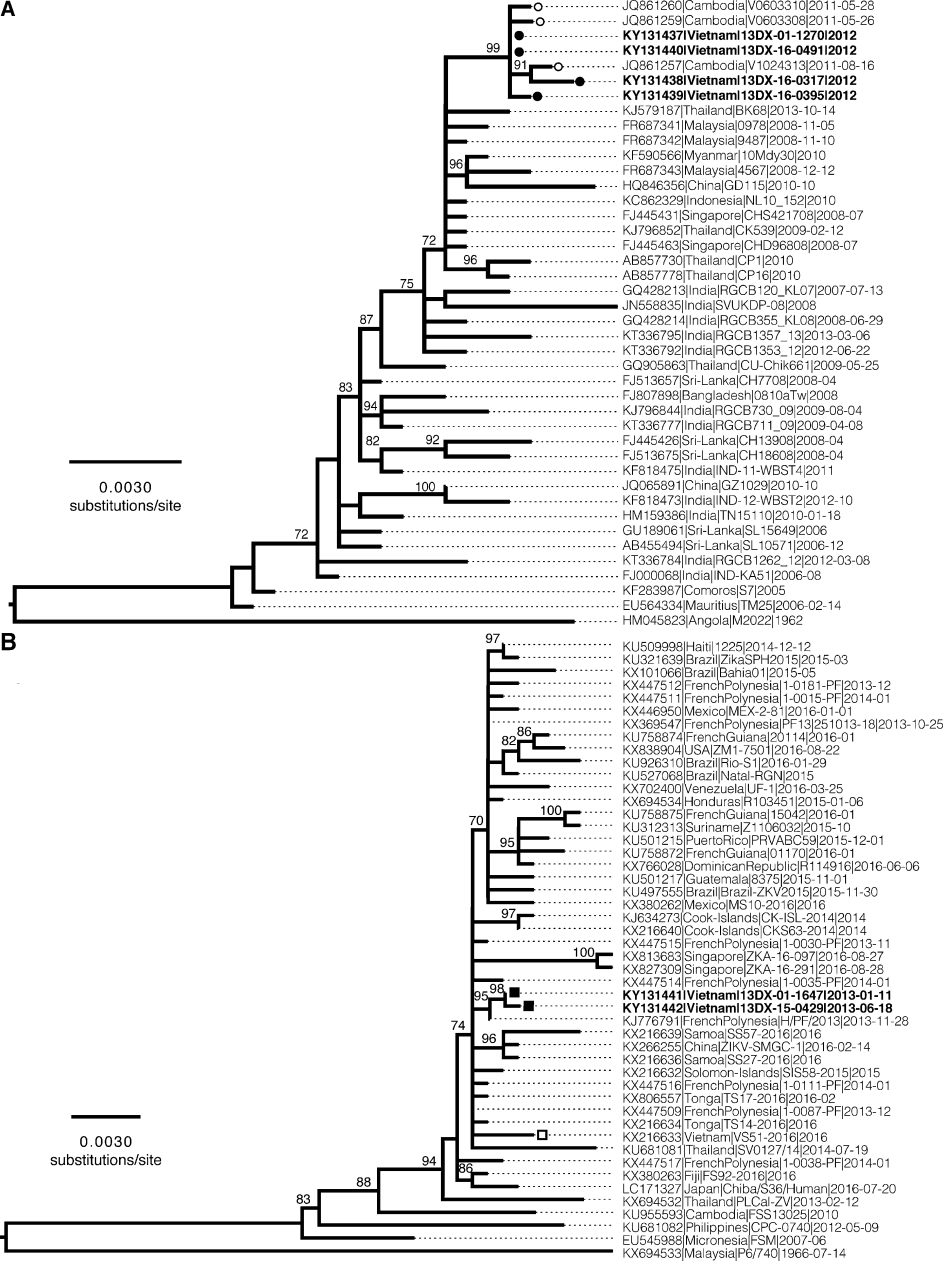
Maximum likelihood phylogenies of chikungunya and Zika virus. (**A**) Maximum likelihood phylogeny of the E1 gene of the chikungunya virus East/Central/South African (ECSA) Indian Ocean Outbreak lineage. The tree was constructed in IQ-TREE (TNe + Γ_4_, 1,000 bootstraps) using a 1,743-nt alignment of a representative subsample of Indian Ocean Lineage (IOL) viruses, with Angola 1962 (HM045823) as an outgroup. Vietnamese isolates are indicated by closed black circles. Cambodian sequences are shown with open circles. (**B**) Maximum likelihood phylogeny of the E gene of the Zika virus Asian genotype. The tree was constructed in IQ-TREE (Vienna, Austria; TN + Γ_4_, 1,000 bootstraps) using a 1,512-nt alignment of a representative subsample of Asian genotype viruses, with Malaysia 1966 (KX694533) as an outgroup. Vietnamese isolates from this study (2013) are indicated by closed black squares. The Vietnamese isolate from 2016 is indicated by an open square. Bootstrap values are shown for nodes with bootstrap support ≥ 70%. Scale bars represent the number of nucleotide substitutions per site.

Nucleotide sequences of the 1,743-nucleotide (nt) CHIKV E1 gene with partial 3′ untranslated region and 1,512-nt ZIKV E gene were determined using Miseq Illumina (San Diego, CA) techniques. Sequence assembly and alignment of these sequences to global databases was performed using CLC Workbench 9.1 (Qiagen, Redwood City, CA). The four Vietnamese CHIKV E1 sequences (GenBank Accession KY131437, KY131438, KY131439, and KY131440) shared 99.77–100% pairwise nucleotide identity and carried the E1-A226V mutation reported to increase infectiousness for *Aedes albopictus*.^16^ Phylogenetic analysis indicated that the four Vietnamese samples were of the Indian Ocean Outbreak lineage within the ECSA genotype and clustered closely with 2011 Cambodian sequences (99.71–99.94% identity) (Figure 2A

). Notably, two of the Vietnamese cases in Binh Duong Province resided approximately 20 km from the Vietnam–Cambodian border.

The two ZIKV E gene sequences captured in Vietnam in 2013 (GenBank Accession KY131441 and KY131442) differed by a single nucleotide. There was strong bootstrap support to place these sequences among other Asian lineage ZIKV sampled from southeast Asia and the Western Pacific, with a close phylogenetic link to a sequence obtained from French Polynesia in late 2013 (Figure 2B). The similarity of the two Vietnamese sequences coupled with their spatial and temporal spread (42 km and 5 months apart), suggests that ZIKV may have circulated at very low levels or caused mild enough illness to be largely undetected within southern Vietnam in 2013. Alternatively, the viruses could represent multiple isolated importations of ZIKV from another population. As this investigation was conducted retrospectively and travel histories were not collected at enrollment, we cannot definitively determine whether the viruses detected in this study were locally acquired or travel related. Importantly, these sequences do not appear to be phylogenetically linked to the only other available ZIKV sequence from Vietnam, sampled in 2016 (GenBank accession KX216633), which suggests that the two 2013 viruses characterized in this study are unlikely to represent ancestral viruses to those causing more recent infections within Vietnam.

## CONCLUSIONS

These data suggest that CHIKV and ZIKV have recently circulated, or been imported, into southern Vietnam. The detection of Zika cases in 2013 is concerning for the potential consequences of local transmission for pregnant women. Greater surveillance is required to understand the trajectory of the incidence of these arboviral diseases in Vietnam, and underscores the importance of performing differential diagnosis in populations where multiple arboviral pathogens may cocirculate.
